# Co-expression of high-molecular-weight glutenin subunit *1Ax1* and *Puroindoline a* (*Pina*) genes in transgenic durum wheat (*Triticum turgidum ssp. durum*) improves milling and pasting quality

**DOI:** 10.1186/s12870-019-1734-x

**Published:** 2019-04-04

**Authors:** Qiong Wang, Yin Li, Fusheng Sun, Xiaoyan Li, Pandi Wang, Junli Chang, Yuesheng Wang, Guangxiao Yang, Guangyuan He

**Affiliations:** 1 The Genetic Engineering International Cooperation Base of Chinese Ministry of Science and Technology, Key Laboratory of Molecular Biophysics of Chinese Ministry of Education, College of Life Science and Technology, Huazhong University of Science and Technology (HUST), Wuhan, 430074 China; 2 College of Life Science and Health, Wuhan University of Science and Technology, Wuhan, 430065 China; 3 Waksman Institute of Microbiology, Rutgers, The State University of New Jersey, 190 Frelinghuysen Rd, Piscataway, NJ 08854 USA

**Keywords:** Durum wheat, Grain hardness, End-use quality, Milling quality, Pasting property, Puroindoline, High-molecular-weight glutenin subunit

## Abstract

**Background:**

Durum wheat is considered not suitable for making many food products that bread wheat can. This limitation is largely due to: (i) lack of grain-hardness controlling genes (*Puroindoline a* and *b*) and consequently extremely-hard kernel; (ii) lack of high- and low-molecular-weight glutenin subunit loci (*Glu-D1* and *Glu-D3*) that contribute to gluten strength. To improve food processing quality of durum wheat, we stacked transgenic *Pina* and HMW-glutenin subunit *1Ax1* in durum wheat and developed lines with medium-hard kernel texture.

**Results:**

Here, we demonstrated that co-expression of *Pina* + *1Ax1* in durum wheat did not affect the milling performance that was enhanced by *Pina* expression. While stacking of *Pina* + *1Ax1* led to increased flour yield, finer flour particles and decreased starch damage compared to the control lines. Interestingly, *Pina* and *1Ax1* co-expression showed synergistic effects on the pasting attribute peak viscosity. Moreover, *Pina* and *1Ax1* co-expression suggests that PINA impacts gluten aggregation via interaction with gluten protein matrix.

**Conclusions:**

The results herein may fill the gap of grain hardness between extremely-hard durum wheat and the soft kernel durum wheat, the latter of which has been developed recently. Our results may also serve as a proof of concept that stacking *Puroindolines* and other genes contributing to wheat end-use quality from the A and/or D genomes could improve the above-mentioned bottleneck traits of durum wheat and help to expand its culinary uses.

**Electronic supplementary material:**

The online version of this article (10.1186/s12870-019-1734-x) contains supplementary material, which is available to authorized users.

## Background

Durum wheat (*Triticum turgidum ssp. durum*; 2n = 28, AABB) is an allotetraploid species that contributes to ~ 7% global wheat production, while bread wheat (*Triticum. aestivum* L.; 2n = 42, AABBDD) belongs to allohexaploid species that dominates wheat production worldwide [1]. Durum wheat has been widely grown in low-rainfall and semiarid regions, including Australia, Canada, India, Mexico, the Middle East, the Mediterranean countries and Northern Africa [2–5]. It is considered as an agronomically vigorous crop with good biotic and abiotic stress tolerance, but unsuitable for making many food products that bread wheat can [6, 7].

The limited culinary application is largely due to two reasons: (i) durum wheat requires specialized mill to process its extremely-hard grains (hardness index, HI, of more than 80); (ii) the milled flour of durum wheat, semolina, has larger flour particle size, higher levels of damaged starch and water absorption compared to bread wheat [8, 9]. Genetically, these inferior qualities of durum wheat can be explained by lack of the D genome. The short arm of chromosome 5D (5DS) contains the grain hardness (*Ha*) locus largely controlling kernel texture in wheat. Two grain-hardness causal genes on the *Ha* locus, *Puroindoline a* and *b* (*Pina* and *Pinb*, respectively) confer soft kernel phenotype when their wild-type alleles are expressed [10–12]. The cause-and-effect relationship between Puroindolines and grain hardness has been well studied by genetics and transgenic approaches in bread and durum wheat, as well as in maize and rice, demonstrating mutations in the *Pina-D1* or *Pinb-D1* genes are related to hard kernel phenotype [13–22]. Additionally, durum wheat is considered to have weaker gluten strength compared with bread wheat as the high-molecular-weight glutenin subunits (HMW-GS) and low-molecular-weight glutenin subunits (LMW-GS) loci *Glu-D1* and *Glu-D3* are absent due to lack of the D genome [7]. Particularly, the HMW-GS loci *Glu-A1* and *Glu-D1* have been shown to positively contribute to enhancing the micro-structures and aggregation of gluten matrix, thus conferring superior rheological properties of wheat dough [23–25].

To improve the food processing quality of durum wheat, soft-kernel durum (soft-Svevo) has been generated by introgression of the *Ha* locus from the soft bread wheat via homoeologous recombination [19, 26, 27]. Development of a series of soft durum wheat lines further demonstrates that the bread-making qualities of the soft-durum lines vary depending on their parental backgrounds and alleles contributing to gluten strength [9]. The soft-kernel durum wheat has similar flour and milling characteristics compared to soft bread wheat (HI < 40). However, no counterpart of hard- or medium-kernel bread wheat (40 < HI < 80) has been achieved in durum wheat via traditional breeding approaches. Previously, we reported the development of *Pina*-overexpressing transgenic durum wheat with medium-hard kernel texture (HI of ~ 80) [22, 28]. Nevertheless, it is yet to be tested whether stacking of genes or alleles favorable for gluten strength from the A or D genome could improve food processing qualities of durum wheat. As a proof-of-concept, we crossed the *Pina*-overexpressing line with a *1Ax1*-expressing transgenic durum line that shared the same genetic background and showed that stacking transgenic *Pina* and *1Ax1* in durum wheat has combined effects on dough mixing parameters and could be useful for breeding durum wheat with dual purpose (for pasta and bread) [21]. Still, it is lacking that: (i) whether stacking of HMW-GS would affect milling attributes in the medium-hard durum lines? (ii) what are the effects of stacking HMW-GS and PINA on gluten protein matrix and other aspects of flour food processing qualities except for the dough mixing property that has been studied before? [21] In addition, PINs are starch granule-bound proteins that could influence starch-protein or starch-lipid interactions and possibly impact on starch-related properties, such as pasting property. Evidence also showed that PINs could be located in storage protein matrix during kernel development [29] and could be involved in modifying gluten protein aggregation via hydrophobic interactions [30]. To address the above questions, in the present study, we particularly focused on the effects of *1Ax1* + *Pina* co-expression on milling characteristics, aggregation pattern of gluten proteins and pasting property.

## Results

### Grain hardness and milling performance

**Fig. 1:**
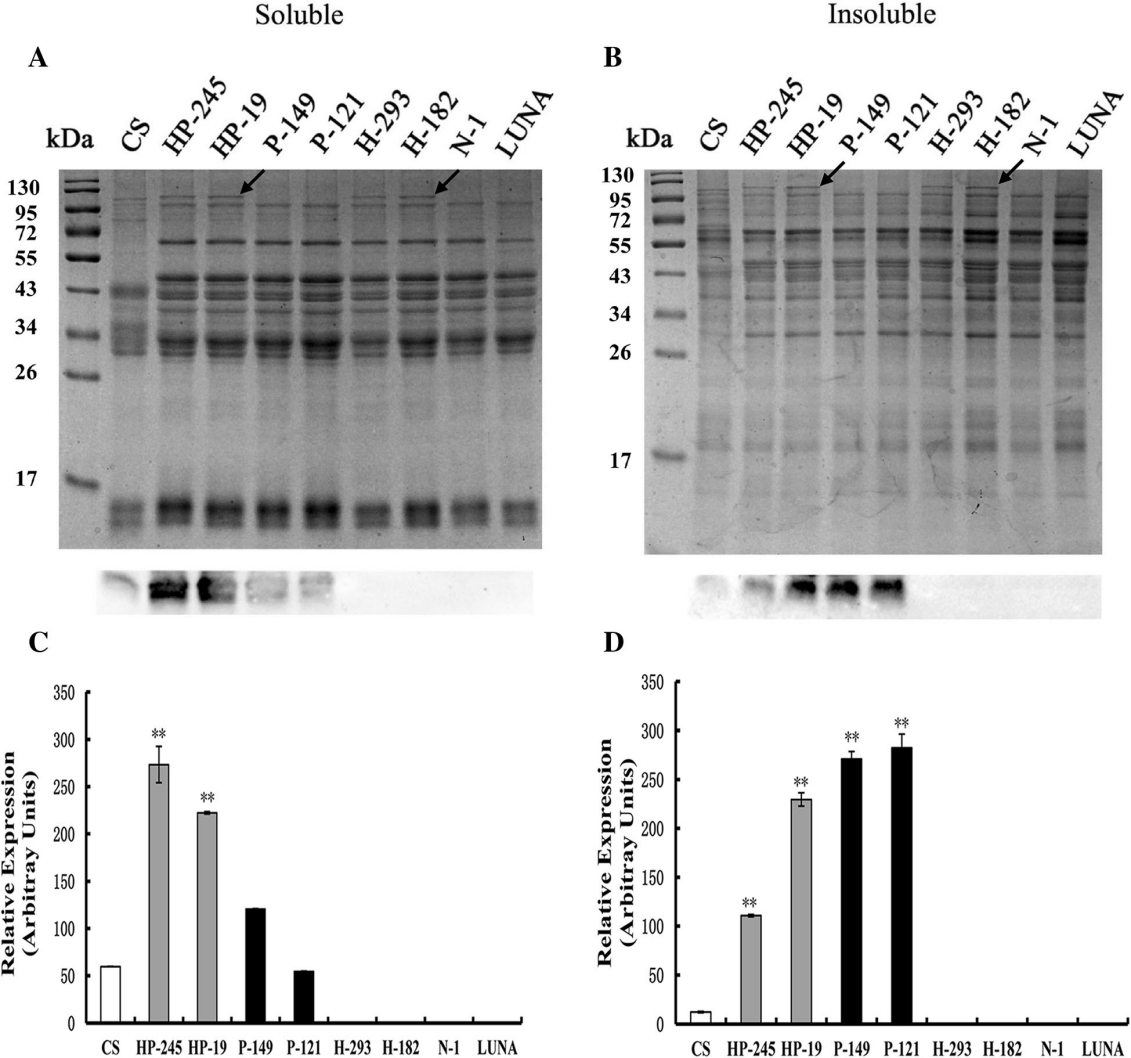
SDS-PAGE and Western blotting analyses of PINA in alcohol-soluble and insoluble proteins extracted from the seeds of transgenic and control lines. **a, c** SDS-PAGE and Western blotting of PINA detected in soluble gluten protein fractions. Transgenic 1Ax1 is indicated by arrowhead. **b**, **d** SDS-PAGE and Western blotting analysis of PINA detected in insoluble gluten protein fractions. ** shows the statistical comparison of PINA abundance between the transgenic lines and positive control line CS. (**P < 0.01 measured by Student’s t-test)

As shown in Table 1, transgenic lines co-expressing *1Ax1* and *Pina* (HP-19 and HP-245) and those only expressing *Pina* (P-121 and P-149) showed similar grain hardness (HI of ~ 60), with the kernel textures of both being softer than those of the control lines (the null-segregant line N-1; the non-transgenic donor Luna; HI > 95) and the *1Ax1* expressing lines (H-182 and H-293 with HI values > 100). The results of kernel texture are consistent with those reported previously [21]. Protein analysis confirmed that HMW-GS *1Ax1* was expressed in lines HP-245, HP-19, H-293 and H-182, while *Pina* overexpression was detected in HP-245, HP-19, P-149 and P-121 (Fig. 1). In accordance with the results of kernel texture, *1Ax1*-*Pina-* co-expressing lines and lines only expressed *Pina* exhibited similar distribution patterns of millstream and significantly increased total flour yield compared to the other lines, improved from ~ 44% (H-182, H-293, N-1 and Luna) to over 60% (Table 1). Compared to *Pina*-absent lines (H-182, H-293, N-1 and Luna), the total flour yield of *Pina*-expressing lines (HP-19, HP-245, P-121 and P-149) was improved due to the increased amounts of break flour and reduction flour, which were raised from ~ 9% and ~ 34% to ~ 17% and ~ 41%, respectively. These results demonstrated that transgenic *1Ax1* itself did not affect milling property and stacking of *1Ax1* and *Pina* transgenes did not influence the improvement of milling performance caused by PINA. It is worth notice that the medium-hard lines of durum wheat had a different millstream distribution pattern compared to that of soft-durum wheat [27]. The soft-durum wheat had grain hardness index of less than 40, with drastically increased break flour yield but slightly decreased reduction flour yield compared to hard durum Svevo. Here, the *Pina* transgenic lines had a medium-hard phenotype with distinct millstream results, which may consequently have different effects on end-use qualities compared with the soft-texture durum lines.

**Table 1:** End-use quality parameters of the transgenic and control lines

Parameters	Line							
	LUNA^a^	N-1^a^	HP-19^a^	HP-245^a^	H-182^a^	H-293^a^	P-121^a^	P-149^a^
Transgene	None	None	*1AX1 + Pina*	*1AX1 + Pina*	*1AX1*	*1AX1*	*Pina*	*Pina*
Hardness^b^	98.70 ± 2.30ab	95.90 ± 5.20b	61.60 ± 1.90 cd	62.90 ± 3.20 cd	105.80 ± 4.20a	101.70 ± 0.60a	60.50 ± 1.50 cd	67.70 ± 20.5c
Flour yield^c^								
Bran(g)	9.15 ± 0.20b	7.50 ± 0.30b	12.26 ± 0.30a	13.25 ± 0.10a	7.61 ± 0.04b	7.54 ± 0.20b	12.42 ± 2.20a	13.95 ± 0.05a
Break flour(g)	9.50 ± 1.30bc	8.60 ± 0.68c	17.36 ± 0.08a	17.48 ± 0.25a	8.83 ± 0.20c	8.95 ± 0.08bc	17.45 ± 0.52a	18.34 ± 0.01a
Shorts(g)	45.87 ± 4.04ab	48.63 ± 2.56a	26.14 ± 0.39c	25.78 ± 0.06c	47.78 ± 0.06ab	48.08 ± 0.83ab	25.25 ± 2.12c	23.95 ± 0.55c
Reduction flour(g)	33.95 ± 2.34b	34.12 ± 1.70b	41.87 ± 0.21a	40.76 ± 0.44a	34.91 ± 0.01b	34.18 ± 1.25b	42.16 ± 0.73a	41.30 ± 0.42a
Total flour(g)	98.47 ± 0.17ab	98.85 ± 0.07a	97.63 ± 0.02b	97.27 ± 0.52b	99.12 ± 0.19a	98.75 ± 0.27a	97.28 ± 0.19b	97.53 ± 0.18b
BFY (%)	9.65 ± 1.33bc	8.70 ± 0.68c	17.78 ± 0.08a	17.96 ± 0.16a	8.90 ± 0.18c	9.07 ± 0.06bc	17.94 ± 0.57a	18.80 ± 0.03a
SFY (%)	44.13 ± 3.77bc	43.21 ± 2.38c	60.67 ± 0.14a	59.87 ± 0.38a	44.12 ± 0.12bc	43.68 ± 1.22bc	61.28 ± 0.10a	61.14 ± 0.54a
Flour characteristics^d^								
Protein content (%)	14.35 ± 0.05a	14.15 ± 0.05a	14.25 ± 0.05a	14.10 ± 0.00a	14.25 ± 0.05a	14.50 ± 0.00a	12.50 ± 0.00b	12.60 ± 0.00b
Water content (%)	12.70 ± 0.00b	12.60 ± 0.00b	12.95 ± 0.05a	13.00 ± 0.00a	12.60 ± 0.00b	12.55 ± 0.05b	13.00 ± 0.00a	13.05 ± 0.05a
Ash content (%)	0.87 ± 0.05b	0.85 ± 0.30b	0.76 ± 0.00c	0.77 ± 0.05c	0.82 ± 0.00b	1.01 ± 0.05a	0.69 ± 0.00d	0.68 ± 0.05d
Gluten content (%)	30.90 ± 0.05bc	30.15 ± 0.30c	33.15 ± 0.00a	32.15 ± 0.05ab	30.7 ± 0.00bc	31.5 ± 0.05b	29.05 ± 0.00d	29.00 ± 0.05d
Color parameters^e^								
L*	77.42 ± 0.23d	79.00 ± 0.32c	81.48 ± 0.52b	82.25 ± 1.69b	79.83 ± 0.19c	80.09 ± 0.38c	82.13 ± 0.44b	84.27 ± 0.12a
a*	−0.98 ± 0.01	−0.80 ± 0.02	− 0.94 ± 0.02	−1.03 ± 0.09	−0.90 ± 0.01	− 0.94 ± 0.02	−0.86 ± 0.03	−1.07 ± 0.01
b*	18.15 ± 0.05a	17.14 ± 0.07ab	13.94 ± 0.09 cd	14.08 ± 0.26c	17.85 ± 0.04a	16.77 ± 0.07b	14.63 ± 0.07c	14.65 ± 0.02c
Damaged Starch (%)^f^	27.60 ± 0.35a	29.70 ± 0.40a	19.20 ± 0.38b	20.20 ± 0.59b	30.60 ± 0.46a	29.50 ± 0.27a	20.50 ± 0.15b	20.00 ± 0.50b
WBC (%)^g^	84.27 ± 0.40b	85.20 ± 0.80b	72.27 ± 0.40c	62.27 ± 1.20d	87.73 ± 0.80b	94.67 ± 0.80a	61.47 ± 0.80d	74.53 ± 1.20c
Flour particle size distribution^h^								
D10(μm)	5.07 ± 0.02a	5.34 ± 0.17a	4.29 ± 0.01b	4.28 ± 0.04b	5.18 ± 0.03a	5.06 ± 0.11a	4.30 ± 0.12b	4.28 ± 0.01b
D50(μm)	26.61 ± 0.12a	26.46 ± 0.02a	25.52 ± 0.55b	25.09 ± 0.16b	26.30 ± 0.09a	26.36 ± 0.03a	25.36 ± 0.14b	24.99 ± 0.05b
D90(μm)	73.21 ± 0.16	71.79 ± 0.66	74.97 ± 3.22	74.67 ± 1.72	73.34 ± 0.37	77.15 ± 3.45	74.53 ± 0.97	70.12 ± 0.38
D43(μm)	33.85 ± 0.07	33.50 ± 0.10	33.57 ± 1.06	33.23 ± 0.47	33.71 ± 0.13	34.85 ± 1.04	33.3 ± 0.31	32.17 ± 0.11
D32(μm)	10.88 ± 0.02a	11.20 ± 0.29a	9.44 ± 0.10b	9.28 ± 0.26b	10.98 ± 0.10a	10.78 ± 0.16a	9.43 ± 0.03b	9.55 ± 0.02b
D21(μm)	2.21 ± 0.01a	2.31 ± 0.10a	1.80 ± 0.10b	1.80 ± 0.10b	2.27 ± 0.03a	2.19 ± 0.04a	1.87 ± 0.01b	1.92 ± 0.01b
SSA(m^2/kg)	177.67 ± 0.39b	172.75 ± 4.45b	205.00 ± 2.10a	206.5 ± 3.60a	175.93 ± 1.40b	179.45 ± 2.65b	205.15 ± 0.75a	202.55 ± 0.35a
RVA attributes ^i^								
Peak viscosity	393.00 ± 16.00d	320.50 ± 8.50e	428.50 ± 11.50c	432.00 ± 2.00c	328.50 ± 16.50e	380.50 ± 22.50d	456.50 ± 2.50b	513.50 ± 16.50a
Setback	47.00 ± 1.00c	30.00 ± 0.00d	37.00 ± 2.00 cd	42.50 ± 6.50c	56.50 ± 10.50b	73.00 ± 8.00a	58.00 ± 8.00b	59.00 ± 3.00b
Breakdown	264.00 ± 6.00d	212.00 ± 9.00e	314.50 ± 4.50c	308.50 ± 1.50c	206.50 ± 6.50e	254.50 ± 12.50d	335.00 ± 4.00b	383.00 ± 17.00a
Trough	129.00 ± 10.00a	108.50 ± 0.70d	114.00 ± 7.00d	123.50 ± 3.50b	122.00 ± 10.00b	126.0 ± 10.00ab	121.50 ± 1.50c	130.50 ± 0.50a
Final viscosity	176.00 ± 11.00c	138.50 ± 0.50f	151.00 ± 5.00e	166.00 ± 10.00d	178.50 ± 20.50c	199.00 ± 18.00a	179.50 ± 6.50c	189.50 ± 2.50b
Peak time (min)	4.25 ± 0.00b	3.85 ± 0.07c	4.09 ± 0.17bc	4.05 ± 0.13bc	3.92 ± 0.20bc	4.55 ± 0.03a	4.02 ± 0.04bc	4.29 ± 0.04ab
Pasting temp(°C)	67.75 ± 4.20	70.65 ± 0.45	56.15 ± 5.70	64.53 ± 7.38	62.63 ± 0.78	65.30 ± 9.00	59.18 ± 1.92	66.40 ± 4.60

Note ^a^Means with the same letter are not significantly different (*P* > 0.05). All data are presented as mean ± SEM

^b^Measured by single kernel characterization system (SKCS) from 300 seeds per line and plot

^c^Measured by Chopin CD1 mill (*n* = 4); *BFY* break flour yield, *SFY* straight-grade flour yield

^d^Measured by near-infrared reflectance spectroscopy (NIRS) method

^e^Measured by Minolta Chroma meter CR-410. L*, lightness; a*, red to green; b*, blue to yellow

^f^Measured by using SDmatic by Chopin Technologies

^g^Measured by the AACC method 56–30

^h^Measured by laser light scattering. D10: the diameter where 10% of the population lies below the D10; D50 (the median): the diameter where half of the population lies below the D50; D90: the diameter where 90% of the distribution lies below the D90; SSA: specific surface area; D21: Length mean diameter; D32: Surface Weighted Mean; D43: Volume Weighted Mean

^i^Measured by rapid visco-analyzer (RVA)

### Flour quality

For flour characteristics, it appears that PINA expression was associated with decreased flour protein content, while 1Ax1 expression was not (Table 1). Flour protein content is a trait that is highly impacted by environment and genotype x environment effects [8, 31, 32]. Moreover, studies on near isogenic lines of durum and bread wheat with or without PINs suggested that PINs may be associated with lowered protein content [33, 34]. The association of PINA with flour protein content needs further investigation. Additionally, the lines only expressed *Pina* showed significantly lower gluten content compared to the remaining lines, while *1Ax1* + *Pina* co-expressing lines had the highest gluten content, possibly due to both the flour protein content and 1Ax1 expression. For flour color, only *Pina* expression was correlated to increased whiteness and decreased yellowness, whereas the 1ines only expressing *1Ax1* did not differ from the control lines in color characteristics (Table 1).

We further analyzed several flour traits for the transgenic and control lines, including flour particle size distribution, water binding capacity (WBC) and starch damage. Starch damage and water binding capacity showed similar trend: the decreased starch damage and WBC were associated with transgenic PINA, with 1Ax1 expression seemingly not related to both traits (Table 1). This result is in line with the positive correlation between grain hardness, starch damage and WBC, as hard-textured grains have stronger adhesion between starch granules and protein matrix and hence produce more damaged starch during milling [35–39].

**Fig. 2:**
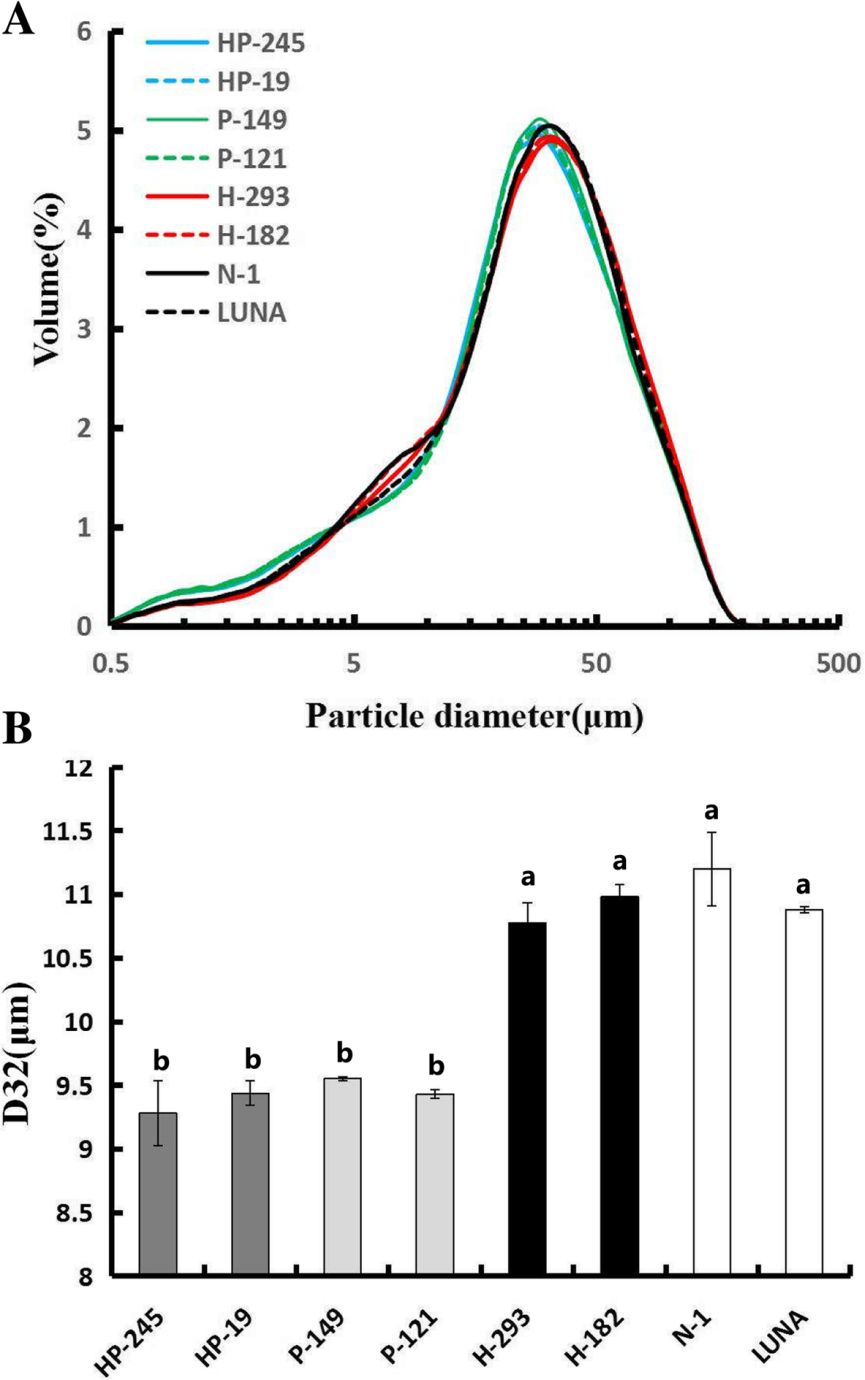
Effects of 1Ax1 and PINA on flour particles size distribution. **a** The size distribution curve of flour particles. **b** Weighted average diameter D32 of flour particles. Data are given as mean ± SEM, calculated from three replicates. The columns labeled by different letters indicate significant difference (*P* < 0.05)

Besides, the lines only expressed *Pina* and those co-expressed *Pina* + *1Ax1* showed similar particle size distribution of straight-grade flour, while the lines only expressed *1Ax1* had similar flour particle size distribution compared with the control lines (Table 1; Fig. 2). These results indicated that flour particle size is determined by grain hardness but not HMW-GS 1Ax1.

### Pasting property determined by rapid visco-analyzer (RVA)

**Fig. 3:**
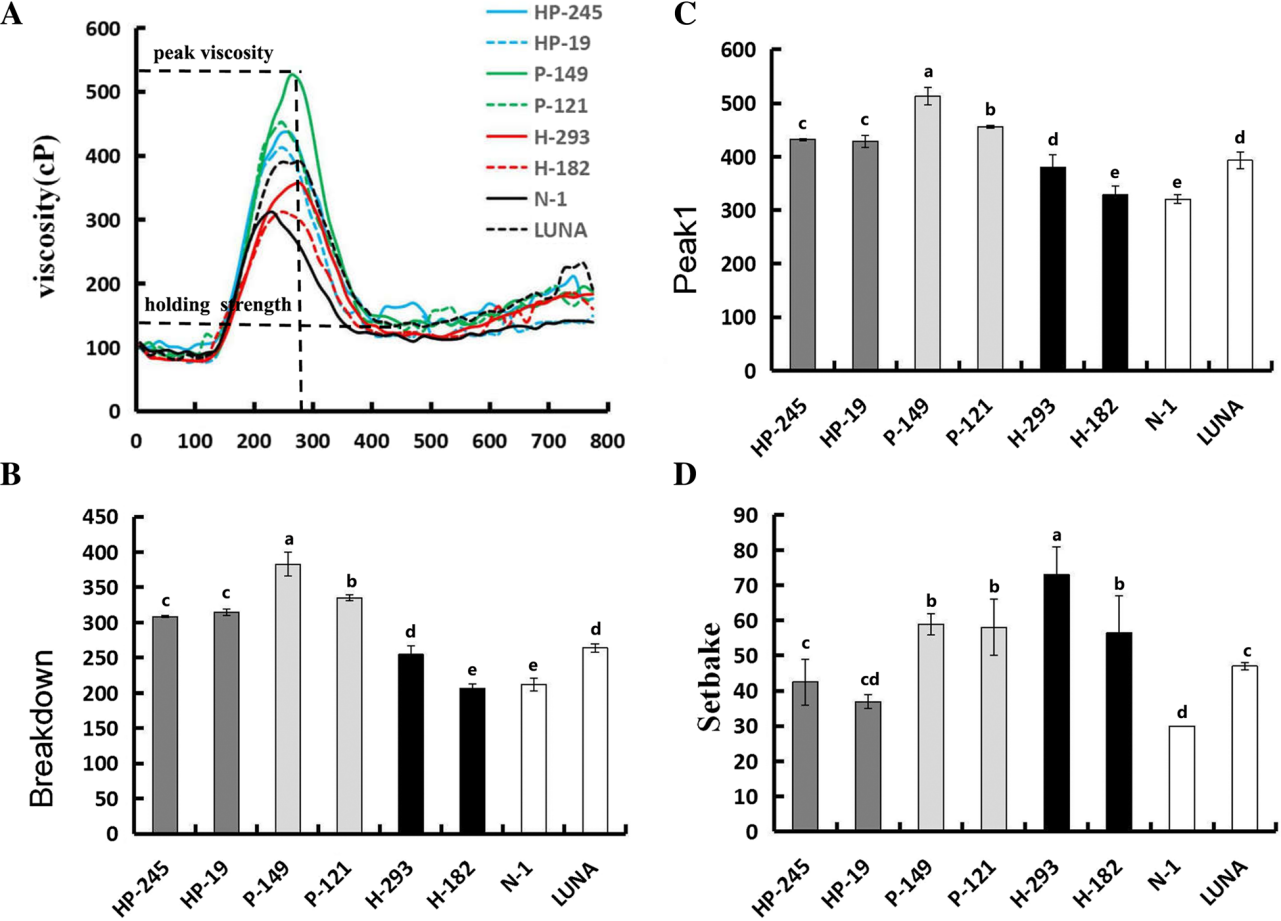
Pasting properties of wheat flours from the transgenic and control lines. **a** RVA pasting profile of the flour samples from transgenic lines and control lines. **b**, **c** and **d** Comparison of three RVA parameters between the transgenic and control lines. Data are given as mean ± SEM, calculated from three replicates. The columns labeled by different letters indicate significant difference (*P* < 0.05)

Interestingly, PINA expression was associated with increased peak and breakdown viscosity, whereas no obvious differences in other RVA attributes were detected between the lines with expression of *Pina, 1Ax1* and *Pina* + *1Ax1* (Table 1; Fig. 3). More importantly, synergistic effects of PINA and 1Ax1 on peak and breakdown viscosity were observed. For example, peak viscosity values of the lines expressed only *Pina*, or *1Ax1*, and co-expressed *Pina* + *1Ax1* were ~ 484, ~ 355 and ~ 430, respectively. The 1Ax1 expression itself did not have effects on RVA attributes. The trough values were similar between the transgenic and control lines (Table 1; Fig. 3). Previous studies also indicated the association between PINA expression and altered pasting parameters in transgenic rice and near isogenic lines of bread and soft-durum wheat, such as peak viscosity and pasting temperature [34, 40]. Particularly, only peak and breakdown viscosity were increased with PINA expression in the present study. Several factors could impact pasting property, including amylose content, amylose/amylopectin ratio, interactions between starch, starch-associated proteins and lipids [41–44]. Several explanations of the PINA- peak viscosity association may be possible: (i) Puroindolines are starch granule-binding proteins and could serve as surfactant to improve the swelling power of starch granules [45]; (ii) Puroindolines may impact on starch interaction with polar lipids since PINs are associated with increased starch bound polar lipids [46]; (iii) A side-effect of decreased grain hardness that softer kernel could be associated with altered physiochemical properties of starch and/or damaged starch granules, thus changing the swelling power; (iv) PINA or PINA-lipid complex may also impact on gluten protein matrix that interacts with starch granules. The fourth explanation appears to match with the synergistic effects of PINA+1Ax1: PINA might play roles in both starch granule surface and protein matrix. Moreover, we separated alcohol soluble and insoluble fractions, the latter of which starch granules may be enriched in. PINA were well detected in both fractions (Fig. 1), indicating transgenically overexpressed PINA might have dual locations as discussed above. Indeed, recent evidence of the subcellular localization of Puroindolines and characterization of protein matrix with or without PINs supported the notion that PINs are located in gluten protein matrix and could interact with some gluten proteins via hydrophobic interactions besides its location of starch granule surface [29, 30].

### Gluten protein aggregation

**Fig. 4:**
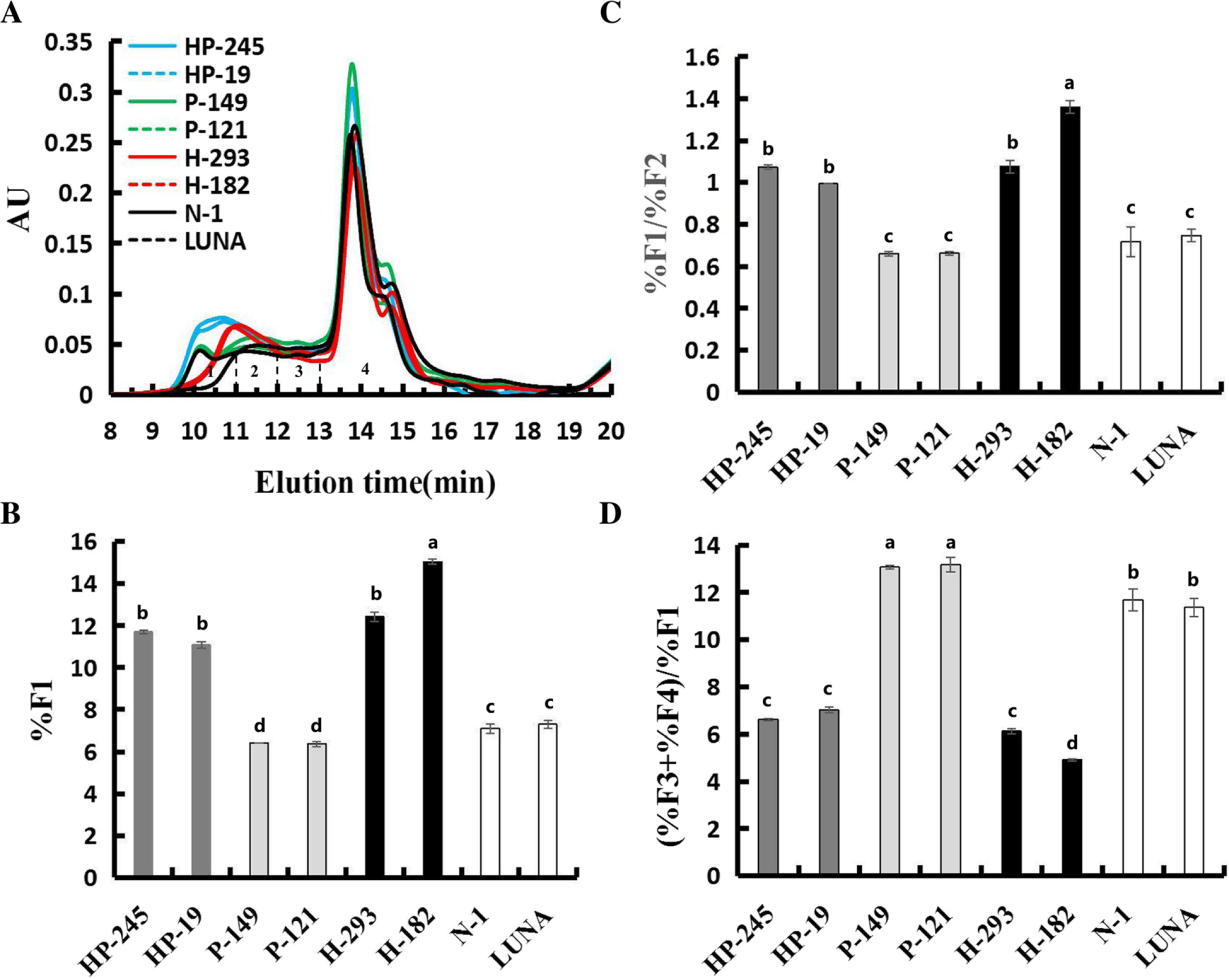
Effect of PINA and 1AX1 on gluten protein aggregation. **a** SE-HPLC analyses of the flour samples from transgenic and control lines. **b**, **c** and **d** Comparisons of three parameters (%F1, %F1/%F2 and (%F3 + %F4)/%F1) between the transgenic and control lines. Data are given as mean ± SEM, calculated from three replicates. The columns labeled by different letters indicate significant difference (*P* < 0.05)

In the present study, SE-HPLC was used to fractionate cold-SDS extracted gluten proteins based on molecular masses without breaking down the inter-chain disulphide bonds [47, 48]. Non-covalent hydrophobic interactions may be dissociated during cold-SDS extraction. The proportion and size distribution of glutenin polymers are important determinants for wheat end-use quality, and accumulated studies have demonstrated that the size distribution of gluten proteins measured by SE-HPLC can serves as a highly reproducible approach to indicate flour functional properties [49, 50]. In Fig. 4, four fractions were detected: large-sized polymers enriched in HMW-GS (F1), medium-sized polymers (F2), oligomers gluten proteins enriched in ω-gliadin (F3), monomeric gluten proteins (mainly α-and γ-type gliadins; F4). Expression of only PINA was associated with increased small monomeric proteins (%F4), while expression of only 1Ax1 was associated with increased polymeric proteins (%F1 + %F2). Moreover, the effects of PINA+1Ax1 were observed as both polymeric and small monomeric proteins were increased (%F1 + %F2, %F4). The relative ratios of several SE-HPLC peaks, namely %F1/%F2 and (%F3 + %F4)/%F1, show strong correlations with dough strength. Generally, %F1/%F2 and (%F3 + %F4)/%F1 are negatively correlated with each other, and %F1/%F2 is positively correlated with the functional parameter G’ (an indication of dough elasticity). Combination of %F1/%F2 and (%F3 + %F4)/%F1 can be used to discriminate the breadmaking quality and dough visco-elasticity between wheat varieties [51–53]. In the present study, PINA overexpression resulted in lowered large size polymer (%F1) and increased (%F3 + %F4)/%F1 compared to the control lines. These negative effects on gluten aggregation and dough visco-elasticity were compensated in the *Pina* + *1Ax1* co-expressing lines (Fig. 4c and d). The ratios and absolute quantities of the four SE-HPLC peaks were clearly reshaped with the expression of PINA and/or 1Ax1 (Fig. 4a). Our results support the hypothesis that PINA interacts with gluten proteins and impacts on gluten aggregation. Previously, Puroindolines were suggestive of changing dough rheological properties and bread parameters based on in vitro reconstitution experiments [54]. Further, it was hypothesized that PINs might interact with gluten proteins by providing some sort of “hydrophobic cores” to modify the structures of gluten aggregates via hydrophobic bonds [30]. Differently, the effects of endogenous PINs in previous study might be less prominent compared to the results shown here, as the transgenically overexpressed PINA are more abundant (Fig. 1). Together with previous studies, the synergistic effect of PINA and 1Ax1 on viscosity reported herein suggests the conclusion that PINs affect several food-processing qualities possibly by interacting with gluten proteins, while the current results do not exclude the possibility that PINs impact on pasting property via their starch/starch-lipid binding ability. Still, it is yet to be established the molecular mechanism of PINs’ interaction with gluten polymers and PINs’ contribution to different food-processing qualities.

## Discussion

In the present study, the effects of PINA and 1Ax1 on milling property, pasting parameters and gluten aggregation have been investigated using the transgenic lines of durum wheat. We show that PINA and 1Ax1 have synergistic effects on the pasting parameters and PINA overexpression can affect gluten protein aggregation (Table 1; Fig. 1). It is worth notice that the *Pina*-overexpression studied herein was driven by the ubiquitin promoter, which provides constitutive expression. Indeed, we detected PINA in both alcohol soluble and insoluble gluten protein fractions (Fig. 1). While the endogenous Puroindolines have been suggestive of being located and possibly exerting functions at both starch granule surface and in gluten protein matrix [29, 30], it was unclear whether the effects of PINA on pasting property and gluten aggregation was due to its ectopic expression or the levels of overexpression. Future studies on PINs’ functionality using transgenic lines with endosperm-specific expression as well as with different PINs expression levels are necessary to further dissect the biological roles on starch-bound PINs and gluten-interacting PINs. In addition, we observed variations in PINA expression levels between transgenic lines (Fig. 1). Similarly, variations of some pasting parameters were found between the null-segregant line N-1 and Luna. Variations in pasting parameters were also observed between the lines expressed *1Ax1* or *Pina* (H-182/H-293, and P-121/P-149, respectively). On one hand, the differences between N-1 and Luna could reflect the variations produced during tissue culture and transgenic process of N-1. The genetic variations could be further accumulated and fixed in the following crossing and multi-generation selfing process when individual transgenic lines expressing *Pina* and/or *1Ax1* were selected [21]. On the other hand, the variations between H-182 vs H-293 or P-121 vs P-149 could be individual differences between transgenic lines. As these transgenic lines were identified among the F_2_ segregants from the cross between a *Pina*-expressing line and a *1Ax1*-expressing line followed by selfing, variations from the two original transgenic lines (expressing *Pina* and *1Ax1*, respectively) could be recombined, fixed and accumulated during multi-generation self-pollination process and therefore obvious differences in some traits were found between individual transgenic lines. Backcross of these transgenic lines to the donor variety Luna for two to three generations and comparison between transgenic and their corresponding null segregant lines would provide more rigorous and robust results to accurately quantify the effects of transgene. It is well known that the contribution of *Glu-1* loci to end-use qualities is *Glu-D1* > *Glu-B1* > *Glu-A1* [55–58]. Although the present and our previous studies have demonstrated the synergistic or additive effects of PINA and 1Ax1 on several aspects of food processing quality, such as dough mixing parameters and pasting parameters [21], it would be valuable to stack *Glu-D1* HMW-GS with PINs through transgenic approaches in durum wheat. Thus, the present study not only provides useful information for quality improvement in durum wheat, but also suggests significant questions that need further investigations.

## Conclusions

Recently, a major advance to expand the culinary use of durum wheat is the development of soft-durum wheat [19, 26, 27, 34, 59, 60]. Further, a series of soft-durum germplasm with varied end-use quality traits have been developed by introgression of the *Ha* locus into different durum wheat varieties. The durum wheat lines with soft-kernel texture represent a paradigm shift in the industrial use of durum wheat grains [8, 9]. Still, these strategies could have a limitation that the favorable genes/alleles for end-use quality traits from the A and D genomes are unable to be stacked to further enhance the food processing qualities of durum wheat. As a proof-of-concept, we stacked *Pina* and *1Ax1* together in durum wheat using transgenic approach and our preliminary results showed improvement in dough mixing quality traits [21]. Here, we demonstrate that stacking of *Pina* and *1Ax1* did not affect the milling performance that was enhanced by PINA expression. Stacking of *Pina* and *1Ax1* in durum wheat lead to medium-hard kernel texture, increased flour yield and decreased starch damage. Importantly, *Pina* and *1Ax1* expression show synergistic effects on pasting property traits and gluten aggregation, suggesting a role of PINA in interaction with gluten protein matrix. The results from the present and several previous studies indicate that the effects of PINs on gluten protein aggregation and different end-use quality attributes need further investigations. Further studies should also examine the effects of PINs in different durum wheat backgrounds.

## Methods

### Plant materials

*1Ax1* or *Pina* transgenic lines of durum wheat was produced in donor variety Luna and described previously [21, 22, 61]. Only HMW-GS pairs 1Bx7 + 1By8 are expressed in Luna [21, 55]. Transgenic *1Ax1* was driven by its endogenous endosperm-specific promoter, while *Pina* was driven by the maize *ubiquitin* promoter. After crossing and selfing of the two transgenic lines, the lines of progeny homozygous for *Pina* (named P-121 and P-149), *1Ax1* (named H-182 and H-293) or *Pina* + *1Ax1* (named HP-19 and HP-245) as well as a null segregant line (N-1) were identified as F_2_ individuals and self-pollinated as described elsewhere [21]. The selection of lines with stable expression of *1Ax1* and/or *Pina* were performed in three continuous generations (F_3_, F_4_ and F_5_) and the F_6_ generation plants were used for measurement of grain hardness and dough mixing property [21]. These lines and their non-transgenic control Luna were grown in the field in 2014 (F_7_ generation) for end-use quality analyses using a randomized complete block design with two replicates in Wuhan (Hubei, China).

### Grain hardness, flour characteristics and flour milling test measurement

Grain hardness were measured with Perten Single Kernel Characterization System (SKCS) 4100 (Perten, Springfield, USA) using the grain samples harvested from each plot according to the AACC approved method 55–31 [62].

Wheat grain samples (100 g per sample) were milled with a Chopin CD1 mill based on the standard method (NF EN ISO 27971:2008; Additional file 1). Flour characteristics were measured by near-infrared reflectance spectroscopy (NIRS) method.

### Determination of damaged starch and water binding capacity (WBC)

Damaged starch content was determined with the amperometric method by using SDmatic by Chopin Technologies (Paris, France; Additional file 1).

The water-binding capacity was measured according to the AACC approved method 56–30 [62] (Additional file 1).

### Color analysis

Flour color parameters L*, a* and b*, which correspond to flour lightness, redness and yellowness, respectively, were evaluated with the CR-410 Chromameter (Minolta, Osaka, Japan) according to Gazza et al. [59].

### Particle size distribution in suspensions of flour

Particle size distribution of wheat flour was evaluated with laser-light scattering particle size analyzer (Mastersizer 2000, Malvern, UK) under a polydisperse analyzing mode and a 300-mm lens. Size distribution was determined in four replications for the flour samples sampled from each field plot (Additional file 1).

### Pasting property determined by rapid Visco-Analyser (RVA)

The flour pasting profile was measured with a Rapid Visco Analyser (RVA-4, Newport Scientific, Australia) (Additional file 1).

### Extraction of soluble and insoluble gluten proteins

Wheat flour was dissolved by 50% (*v*/v) propan-1-ol in a ratio of 10 μL/mg for 30 min. The soluble gluten proteins were obtained by the suspension centrifuged for 5 min at 14,000 g. The insoluble polymeric glutenin proteins were extracted from the residue by using a loading buffer in a ratio of 10 μL/mg. The extracts of soluble and insoluble gluten proteins were analyzed by SDS-PAGE with 10% separating gels and Western blotting with a PINA-specific antibody [22, 47, 63].

### Size exclusion high-performance liquid chromatography (SE-HPLC) analysis

Wheat flour was dissolved by 1 mL of 0.05 M sodium phosphate buffer (pH 6.9) with 0.5% (*w*/*v*) SDS. The flour protein was extracted by sonication for 15 s and centrifuged for 10 min at 13,000 g. The total protein extracts was filtered through a 0.22-μm PVDF membrane and fractionated by using a Waters high-performance liquid chromatography (HPLC) system. According to Tosi et al. [47], four fractions were used to analyze the chromatographic profiles, F1 (large-sized polymers), F2 (medium-sized polymers), F3 (oligomers glutenin) and F4 (monomeric glutenin and other small non-gluten proteins).

### Statistics analysis

All the above-mentioned flour tests, measurements of end-use quality attributes and SE-HPLC analysis were measured in three replicates for each plot. Data obtained from the two replicated plots were used for statistical analysis. Data were calculated using analysis of variance (ANOVA) and significant difference between lines were determined using the least significant difference pairwise comparison and displayed by letters (*p* < 0.05). Data were expressed as mean ± SEM. The statistical significance for western blotting from lines expressing *1Ax1* and/or *Pina* was determined using Student’s *t* test (*p* < 0.05).

## Additional file

Additional file 1: Supplementary materials and methods. (DOCX 24 kb)

## Abbreviations

AACC: American Association of Cereal Chemists
ANOVA: analysis of variance
DAD: photodiode array detection
Ha: Hardness
HI: hardness index
HMW-GS: high-molecular-weight glutenin subunit
HPLC: high-performance liquid chromatography
LMW-GS: low-molecular-weight glutenin subunit
NIRS: near-infrared reflectance spectroscopy
Pina: Puroindoline a
Pinb: Puroindoline b
RVA: rapid visco-analyzer
SDS-PAGE: sodium dodecyl sulfate polyacrylamide gel electrophoresis
SE-HPLC: size exclusion high-performance liquid chromatography
SEM: standard error of the mean
SKCS: single kernel characterization system
WBC: water binding capacity
