# Supplementary material for: Co-expression of high-molecular-weight glutenin subunit 1Ax1 and Puroindoline a (Pina) genes in transgenic durum wheat (Triticum turgidum ssp. durum) improves milling and pasting quality
Source: BMC Plant Biol. 2019 Apr 4;19:126. doi: 10.1186/s12870-019-1734-x (PMC6449967; doi:10.1186/s12870-019-1734-x)
Supplement: Supplementary file 1 — Supplementary materials and methods. (DOCX 24 kb) [file 12870_2019_1734_MOESM1_ESM.docx]

**Supplementary Materias and methods**

1. **Flour milling test measurement**

Wheat grain sample (100 g per sample) was tempered to 16% (w/w) moisture content for 24 h (including 1-h agitation) before milling. Wheat grain sample was milled with a Chopin CD1 mill according to the standard procedure (NF EN ISO 27971:2008). The fraction is then sifted through a series of screens to obtain bran, middlings and break flour. The middlings were then milled with a Chopin CD1 mill again to obtain bran, shorts and reduction flour. Percentage of flour yield (also known as straight-grade flour yield, SFY) was calculated from dividing the flour (break flour plus reduction flour) by the total product (flour, bran, and shorts). Percentage of break flour yield was also calculated. Flour characteristics including flour protein content, flour water content and flour ash content were measured by near-infrared reflectance spectroscopy (NIRS) method using an Infratec TM1241 Grain Analyzer (Foss North America, Silver Spring, MD, USA).

1. **Determination of damaged starch and water binding capacity (WBC)**

Damaged starch is considered to be an important flour quality parameter because it directly affects water absorption. Damaged starch content was determined by SDmatic by Chopin Technologies according to Medcalf & Gilles (1965)[1]. The measuring principle is based on the absorption of iodine by damaged starch in a diluted flour suspension. The more the starch is damaged, the more the iodine is absorbed. The amount of iodine that is absorbed is measured with an amperometric method. A portion of 1 g flour sample was put into the small bucket with 120 mL [solvent](app:ds:solvent) (3 g of boric acid, 3 g of potassium iodine, 120 mL purified water and 1 drop of 0.1M sodium thiosulphate) added in the reaction cup. After the reaction cup was automatically warmed up to 35℃,flour samples were fallen into the reaction cup and subject to measurement. Results were reported in absorbed iodine (AI %) calculated and expressed in UCD unit (Unité Chopin Dubois).

The water binding capacity was recorded as the maximum amount of water that 1 gram of flour sample could retain under low speed centrifugation, according to the procedure given in AACC method 56-30 at ambient conditions [2] . A portion of flour sample (5 g) was weighed and put into a 50-mL centrifuge tube in which 30 mL water was added. The flour-water mixture was stirred for 5 min followed by standing for 30 min at 25℃. The flour-water mixture was then centrifuged at 2000 g for 10 min and the weight of free liquid was measured. The retained weight was expressed as the amount of water absorbed per gram of sample on dry weight basis.

1. **Particle size distribution** **in suspensions of ﬂour**

Particle size distribution of wheat flour was evaluated with laser-light scattering particle size analyzer (Mastersizer 2000, Malvern, UK) under a polydisperse analyzing mode and a 300-mm lens. Size distribution was determined in four replications for the flour samples from each field plot. Flour samples were dispersed in isopropyl alcohol in the equipment circulation unit to attain an obscuration of 15-20%. Before measuring, the sample was circulated in the equipment with mechanical agitation and ultrasound for 1 min to dissolve the ﬂour clots. Measurements were taken at 2 min intervals. Results can be displayed on a volume, surface area, or number basis. The most common approach for expressing laser diffraction results is to report the D10, D50, and D90 values based on a volume distribution. The D50, the median, has been defined above as the diameter where half of the population lies below this value. Similarly, 90 percent of the distribution lies below the D90, and 10 percent of the population lies below the D10. Also, the surface area and volume mean can be used to define the central point. The parameters for defining the volume mean are shown below. SSA, D21, D32 and D43 which has been defined as specific surface area, length mean diameter, surface weighted mean, and volume weighted mean, respectively.

1. **Rapid visco-analysis (RVA)**

The flour pasting proﬁle was measured with a Rapid Visco Analyser (RVA-4, Newport Scientiﬁc, Australia) [3]. A portion of 3.5-g ﬂour and 25-g water were mixed in an aluminum cylindrical bowl. Temperature variation program was applied to the samples during measurement, in which the temperature was first increased from 25℃ to 95℃ over 6 min, maintained constant for 3 min at 95℃ and then decreased from 95℃ to 25℃ over 7 min. When flour was tested in the RVA, the most important parameters measured include pasting temperature, peak time, peak viscosity, breakdown, final viscosity and setback. All the results are expressed in RVA units.

**Supplementary References**

1. Medcalf DG, Gilles KA. Determination of starch damage by rate of iodine absorption. Cereal Chemistry. 1965; 42: 546-557.
2. AACC. American Association of Cereal Chemists Approved Methods, 10th edn. The Association, St Paul. 2000.
3. Collar C. Significance of viscosity profile of pasted and gelled formulated wheat doughs on bread staling. European Food Research and Technology. 2003; 216:505-513.
